# Perspective in radiofrequency ablation for benign thyroid nodules: A perspective

**DOI:** 10.1097/MD.0000000000044748

**Published:** 2025-12-12

**Authors:** Zhuan-ning Han, Xu-ping Shen, Rong-rong Wang

**Affiliations:** a Department of Ultrasound, The Second Affiliated Hospital of Xi’an Medical College, Xi’an, China; b Department of Ultrasound, The First People’s Hospital of Tongxiang, Tongxiang, China; c Department of Function, Xi’an Hospital of Traditional Chinese Medicine, Xi’an, China.

**Keywords:** Benign thyroid nodules, complication, perspective, radiofrequency ablation, safety, treatment

## Abstract

Radiofrequency ablation (RFA) represents a paradigm shift in the minimally invasive management of benign thyroid nodules (BTNs). By utilizing high-frequency radio waves to generate thermal energy, RFA precisely targets and ablates pathological thyroid tissue, offering a substantial improvement over conventional surgical methods. The procedure minimizes trauma, expedites patient recovery, and significantly reduces postoperative complications. Robust empirical evidence supports RFA’s efficacy in reducing BTNs volume while preserving the structural and functional integrity of the surrounding thyroid parenchyma. Technological advancements, such as real-time ultrasound guidance, have enhanced both the precision and safety of RFA procedures. Despite these advancements, gaps remain in the understanding of RFA’s optimal implementation. Further research is needed to refine best practices, evaluate long-term therapeutic outcomes, and establish clearer patient selection criteria. The ongoing advancements in RFA not only improve procedural accuracy but also promise better clinical outcomes, thereby increasing its accessibility and comprehension among healthcare providers and patients. This potential for broader clinical adoption positions RFA as a viable mainstream treatment for BTNs. Anticipated future directions encompass the development of innovative RFA devices, the refinement of therapeutic protocols, and the integration of RFA into multidisciplinary treatment models aimed at enhancing efficacy and safety for patients with BTNs.

## 1. Introduction

Benign thyroid nodules (BTNs) are a common endocrine disorder affecting millions worldwide.^[1]^ While most BTNs are asymptomatic and managed conservatively, some require intervention due to size, location, or symptoms such as dysphagia, dyspnea, or cosmetic concerns.^[1]^ Conventional treatments such as surgery and radioactive iodine therapy are effective but may not be suitable for all patients due to associated side effects and health risks.^[2,3]^ Pretreatment cytological evaluation using fine-needle aspiration or core-needle biopsy is essential to differentiate benign from malignant thyroid lesions.^[2,4]^ This step helps guide appropriate treatment decisions and reduces unnecessary invasive procedures.^[2]^

To address these limitations, radiofrequency ablation (RFA) has emerged as a promising minimally invasive treatment for BTNs.^[3]^ Compared to surgery, RFA offers faster recovery, fewer complications, and lower postoperative morbidity, making it an appealing option for many patients.^[3,5,6]^ Ultrasound guidance enhances procedural accuracy by enabling real-time visualization of the nodule during ablation, reducing the risk of complications.^[7,8]^ However, the use of RFA for BTNs still raises questions about best practices, long-term efficacy, and appropriate patient selection.

This study aims to evaluate the development of ultrasound-guided RFA for BTNs and synthesize current evidence to inform future research and clinical applications. Despite progress in BTNs treatment, current methods still have limitations in scope and understanding. This study addresses key knowledge gaps by analyzing the evolving role of RFA in minimally invasive treatments. It reviews established evidence on RFA’s safety and efficacy, and explores recent technological advances and procedural improvements. By highlighting these advances, this study aims to support clinicians in optimizing RFA to improve patient outcomes. It also emphasizes the potential for integrating RFA into personalized and multimodal treatment strategies to expand its role in BTNs management.

## 2. Radiofrequency ablation: principles and applications

### 2.1. Principles of RFA and its application to BTNs

RFA uses high-frequency alternating current to generate localized heat that precisely ablates targeted thyroid tissue.^[6]^ Advances in electrode design, ultrasound guidance, and procedural techniques have significantly improved RFA’s safety and efficacy. These developments enable accurate control of ablation zones and reduce damage to surrounding healthy tissue.^[9]^ As a result, RFA is now used for a wider range of thyroid lesions. This section reviews recent RFA innovations and their clinical relevance, focusing on improved outcomes and reduced risks.^[8]^ RFA is widely recognized as effective for treating both functional and nonfunctional benign thyroid nodules. Functional nodules produce excess thyroid hormones, potentially causing hyperthyroidism, while nonfunctional nodules may cause compressive symptoms or aesthetic concerns. Unlike surgery, RFA is minimally invasive, allowing faster recovery and fewer post-procedural complications.^[6]^ Its precision in targeting nodules also helps preserve surrounding thyroid tissue and maintain thyroid function.^[6]^

### 2.2. Development of RFA technology and current technical standards

The evolution of RFA from experimental use to standard clinical practice represents a major advance in thyroid nodule treatment. This progress is largely due to improvements in imaging, especially ultrasound, which have improved RFA accuracy and safety. RFA has progressed from basic approaches to more advanced techniques. A major advancement is the moving-shot technique developed by JH Baek.^[10,11]^ This method enables continuous electrode movement during ablation, ensuring uniform energy distribution.^[10,11]^ Earlier devices often led to incomplete ablation and uneven heating, reducing treatment effectiveness. The moving-shot technique addresses these issues by enabling consistent energy delivery, improving treatment efficacy.^[10,11]^ This technique has helped establish RFA as a leading minimally invasive alternative to thyroid surgery. These improvements have made RFA a core non-surgical option with high precision and low risk. Comprehensive guidelines have been developed to ensure the safe and effective use of RFA.^[8,12]^ They include indications, contraindications, procedural methods, and postoperative care. Following these protocols helps ensure patient safety and optimal outcomes. RFA technology continues to evolve through ongoing innovation. Research focuses on improving electrode design and optimizing energy delivery to enhance efficiency and outcomes.^[13]^ Combining RFA with other techniques, such as laser or microwave therapy, may further improve results. As knowledge of thyroid pathology grows, RFA’s clinical applications continue to expand, enhancing patient care.^[14]^

## 3. Clinical research advance of RFA in the treatment of BTNs

**Figure 1.**
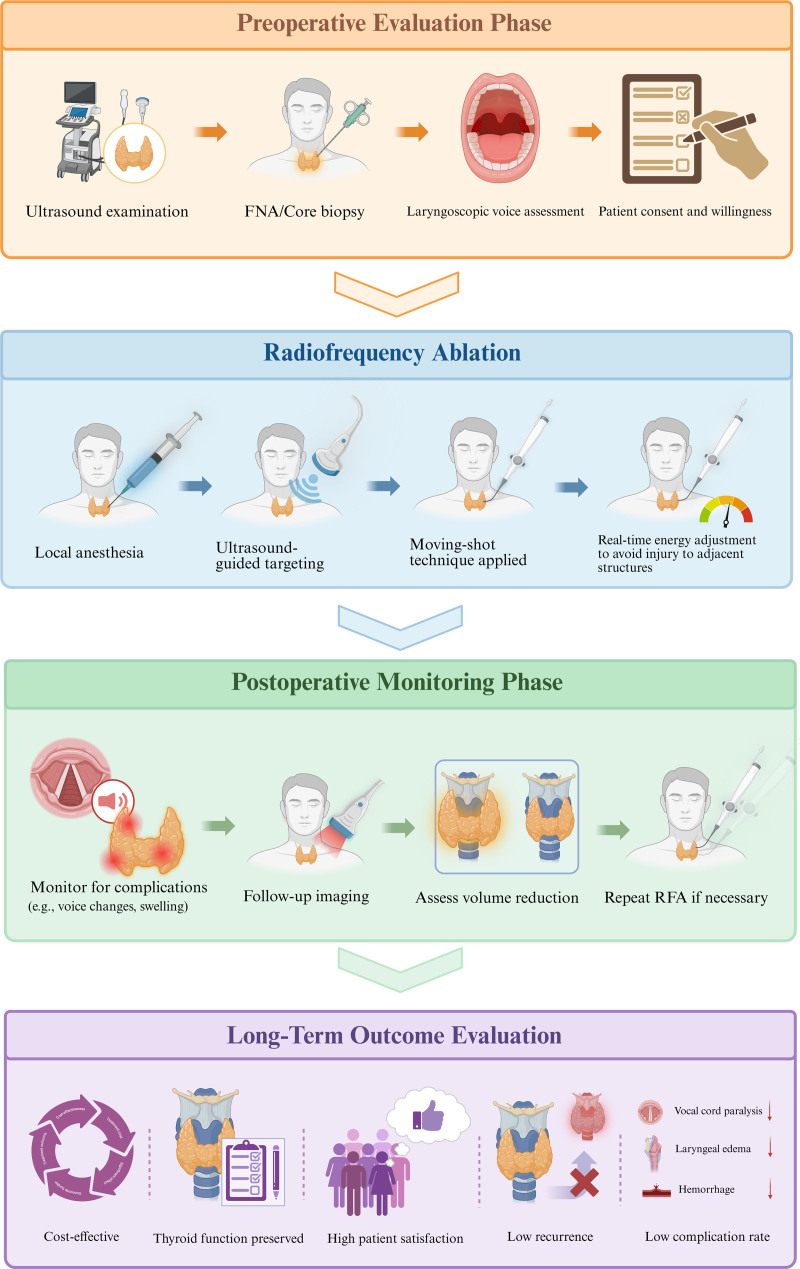
Clinical application flowchart of RFA for BTNs.

Recent advancements in minimally invasive therapies have positioned RFA as a central treatment option for BTNs^[15–25]^ (Table 1). This study summarizes recent clinical research progress on RFA for BTNs by compiling key findings, evaluating its efficacy and safety, and identifying critical factors that influence therapeutic outcomes. By systematically synthesizing current evidence, this study provides an in-depth perspective on the role of RFA in managing BTNs, compares it with other therapeutic options, and highlights areas requiring further investigation (Fig. 1).

**Table 1:** Clinical trial summary of radiofrequency ablation for patients with benign thyroid nodules.

Study Ref.	Year	Comparison	Publication type	Main findings
Zhang et al^[15]^	2024	RFA vs. Microwave Ablation	Randomized Controlled Trial	RFA is effective for solid benign thyroid nodules, with comparable efficacy to microwave ablation.
Schalch et al^[16]^	2021	RFA vs. Thyroidectomy	Prospective Study	RFA is cost-effective compared to conventional thyroidectomy for treating benign thyroid nodules.
Cesareo et al^[17]^	2021	RFA vs. Laser Ablation	Randomized Trial	At 12 months, RFA and laser ablation showed similar efficacy, with RFA having potential advantages in specific cases.
Deandrea et al^[18]^	2015	RFA vs. Observation	Randomized Controlled Trial	RFA demonstrated superior efficacy and safety compared to observation for nonfunctioning benign thyroid nodules.
Turtulici et al^[19]^	2014	RFA	Technical Report	The use of a virtual needle tracking system enhances the precision, safety, and efficacy of RFA.
Zhu et al^[20]^	2020	Anhydrous Ethanol Use in RFA	Randomized Controlled Trial	Efficiency of RFA for treating benign thyroid nodules is improved with the use of anhydrous ethanol.
Cesareo et al^[21]^	2020	RFA vs. Laser Ablation	Randomized Trial	Six-month outcomes show comparable efficacy between laser ablation and RFA for benign nonfunctioning thyroid nodules.
Sung et al^[22]^	2013	RFA vs. Ethanol Injection	Randomized Study	RFA is more effective than ethanol injection for the treatment of benign cystic thyroid nodules in a single-session treatment.
Jin et al^[23]^	2021	Thermal Ablation vs. Surgery	Comparative Study	Patients treated with thyroid thermal ablation, including RFA, reported better postoperative quality of life and satisfaction than those undergoing conventional thyroidectomy.
Hong et al^[24]^	2015	RFA for bilateral benign thyroid nodules	Clinical Study	RFA is a thyroid function-preserving treatment that is effective for patients with bilateral benign thyroid nodules.
Huh et al^[25]^	2012	Multiple RFA Sessions	Randomized Study	Additional RFA treatment sessions increase the efficacy for symptomatic benign thyroid nodules.

BTN = benign thyroid nodules, RFA = radiofrequency ablation.

Although minimally invasive techniques have revolutionized BTNs treatment, uncertainties persist regarding the long-term outcomes and individualized application of RFA.^[26]^ This study enhances understanding of RFA’s clinical utility by assessing key factors, including nodule size, location, and composition, that significantly influence treatment efficacy. For example, smaller nodules (<4 cm) have shown better responses to RFA, highlighting the importance of appropriate patient selection.^[26]^ Furthermore, combining RFA with other ablative techniques such as microwave ablation (MWA) represents a promising strategy for enhancing treatment outcomes in BTNs. By integrating recent findings,^[12]^ this study offers clinicians a comprehensive framework for personalizing RFA protocols, thus optimizing patient outcomes.

### 3.1. Summary of main research findings on RFA for BTNs

Recent studies highlight the efficacy of RFA in significantly reducing thyroid nodule volume, while maintaining a favorable safety profile.^[15–25]^ Although current evidence strongly supports RFA for BTNs management, enhancing its accessibility in clinical settings remains a key challenge. Simplifying procedural protocols and improving patient education could promote broader adoption of RFA and help establish it as a standard treatment modality for BTNs.^[3]^ A notable randomized controlled trial comparing MWA and RFA for solid or predominantly solid BTNs confirmed both methods’ effectiveness in volume reduction^[15]^ (Table 1). However, RFA demonstrated a better safety profile and was associated with higher patient satisfaction.

Although most studies have affirmed RFA’s safety and efficacy, there remains a lack of research exploring novel applications and interdisciplinary integration. The current literature is largely descriptive, with limited emphasis on exploring innovative uses or synergistic integration with other technologies. This study reaffirms existing findings while emphasizing the need to explore combinatorial approaches, such as integrating RFA with laser ablation (LA) or MWA, to improve therapeutic outcomes.^[12,15,17]^ Moreover, the rise of personalized medicine presents opportunities to customize RFA protocols based on patient-specific characteristics, potentially enhancing both short- and long-term outcomes.^[12]^ Investigating these new directions could further establish RFA as a core component of next-generation treatment strategies for BTNs.^[13]^

This review adds to the growing evidence supporting minimally invasive methods like RFA as safe and effective alternatives to surgery, especially for patients with higher surgical risks.^[26]^ By reducing complications and accelerating recovery, RFA aligns with contemporary preferences for minimally invasive interventions, further reinforcing its role in modern BTNs management.^[3]^

A prospective cost-effectiveness analysis revealed that RFA is a more economical alternative to traditional thyroidectomy, offering substantial cost savings without compromising therapeutic efficacy^[16]^ (Table 1). These findings underscore RFA’s value as a cost-effective option, particularly for healthcare systems seeking to optimize clinical outcomes while maintaining financial sustainability.

The LARA II study, a 12-month comparison of LA and RFA, found both techniques effective in reducing nodule volume and improving patient quality of life. However, RFA was linked to shorter procedure durations and greater patient satisfaction, highlighting its practical advantages over LA^[17]^ (Table 1).

### 3.2. Factors influencing the outcome of RFA

The initial size of the thyroid nodule plays a critical role in determining the effectiveness of RFA. Studies have shown that nodules smaller than 4 cm tend to experience greater volume reduction after RFA treatment. This finding is supported by research from Deandrea M et al., who reported notable volume reductions in small, nonfunctioning BTNs following RFA^[18]^ (Table 1).

RFA efficacy is also significantly affected by the anatomical location of the nodule within the thyroid gland. Nodules located near the gland surface or away from critical structures, such as the recurrent laryngeal nerve (RLN), can be ablated more safely and effectively. Technological advances such as virtual needle tracking systems have improved procedural precision in treating difficult-to-access nodules, as demonstrated by Turtulici et al.^[19]^ (Table 1).

The internal composition of thyroid nodules is another important factor affecting RFA outcomes. Solid or predominantly solid nodules typically respond more favorably to RFA, whereas those with substantial cystic components may require adjunctive interventions. Zhu et al found that adding anhydrous ethanol can enhance RFA efficacy in treating cystic nodules^[20]^ (Table 1).

Beyond size, location, and composition, a key determinant of RFA success is the precise calibration of energy delivery during the procedure. Ablation efficacy is directly influenced by the energy applied, which affects the extent of tissue necrosis and the resulting nodule volume reduction. Larger or denser nodules typically require higher energy doses to achieve sufficient tissue penetration and effective ablation. However, excessive energy can elevate the risk of complications, including thermal injury to adjacent structures or surrounding vital tissues. Thus, precise energy modulation is essential to maximize therapeutic efficacy while minimizing procedural risks. Baek et al emphasized the importance of individualized energy protocols, customized according to each patient’s nodule characteristics, to enhance therapeutic outcomes while protecting surrounding structures.^[10,11]^ These findings highlight the need to incorporate precise energy control into standard RFA protocols to achieve optimal clinical outcomes.

## 4. Comparison of RFA with other treatment modalities

RFA has been recognized as a notable advancement in the treatment of BTNs, providing a minimally invasive alternative to conventional surgical and non-surgical therapies. This section presents comparative analyses of RFA and other treatment modalities, including MWA, LA, thyroidectomy, and ethanol ablation (EA), based on current research evidence (Table 2).

**Table 2:** Summary of comparison between RFA and other treatment modalities.

Parameter	RFA	Thyroidectomy	Microwave ablation	Laser ablation	Ethanol ablation
Invasiveness	Minimally invasive	Highly invasive	Minimally invasive	Minimally invasive	Minimally invasive
Anesthesia	Local anesthesia	General anesthesia	Local anesthesia	Local anesthesia	Local anesthesia
Thyroid Function	Preserved	Often lost (lifelong hormone needed)	Preserved	Preserved	Preserved
Hospital Stay	Outpatient procedure	Inpatient stay (longer recovery)	Outpatient	Outpatient	Outpatient
Complication Rate	Low (e.g., RLN injury: 0.2–1.0%)	Higher (e.g., RLN injury: 2–5%)	Comparable to RFA	Slightly higher than RFA	Variable
Cost-Effectiveness	High	Low	Moderate	Moderate	High
Volume Reduction	Significant (esp. <4 cm nodules)	Complete removal	Significant	Effective	Effective for cystic nodules
Patient Satisfaction	High	Variable	High	Moderate	Moderate
Limitations	Requires expertise, no histology	High surgical risk, hypothyroidism	Heat-sink effect in large nodules	Slower volume reduction	Not suitable for solid nodules

BTNs = benign thyroid nodules, RFA = radiofrequency ablation, RLN = recurrent laryngeal nerve.

### 4.1. RFA compared to other modalities

RFA versus thyroidectomy: Schalch MS et al highlight RFA’s potential cost-effectiveness compared with conventional thyroidectomy, especially when considering long-term health outcomes and economic impact. This benefit reflects RFA’s less invasive nature and its ability to reduce the broader complications associated with surgery.^[13]^

RFA versus MWA: Zhang et al report that both RFA and MWA are effective treatment options, and the choice between them may depend on nodule characteristics and individual patient preferences. This finding underscores the importance of personalized treatment planning.^[12]^

RFA versus LA: The 12-month LARA II study by Cesareo et al reveals differences in efficacy, safety, and patient satisfaction between LA and RFA, highlighting the need for further studies to clarify these differences.^[14,18]^

RFA versus EA: Sung et al report that RFA provides more consistent volume reduction, particularly in cystic or predominantly cystic nodules, indicating a potential advantage over EA in selected cases.^[19]^

### 4.2. Advantages of RFA

Minimally invasive nature: RFA’s less invasive approach leads to reduced postoperative pain and minimal scarring. Jin et al documented improved quality of life and higher patient satisfaction with thermal ablation, including RFA, compared to more invasive procedures.^[20]^

Preservation of thyroid function: Hong et al demonstrated that RFA can preserve normal thyroid function, which is a key advantage over total thyroidectomy that typically requires lifelong hormone replacement due to hypothyroidism.^[21]^

Efficacy in nodule reduction: Deandrea et al emphasized RFA’s efficacy in significantly reducing the volume of nonfunctioning BTNs, reinforcing its value as a non-surgical treatment option.^[15]^

Economic and logistical benefits: As an outpatient procedure, RFA enables shorter hospital stays and reduced overall treatment costs. Schalch et al emphasized its cost-effectiveness compared with thyroidectomy, considering both direct and indirect healthcare savings.^[13]^

### 4.3. Disadvantages of RFA

Risk of incomplete treatment: RFA may require multiple treatment sessions to achieve complete ablation, particularly in larger or complex nodules. Huh et al noted this limitation in their evaluation of RFA’s multi-session efficacy for symptomatic BTNs.^[22]^

Dependence on technical expertise: RFA outcomes largely depend on the operator’s skill and experience, which may result in variability across different clinical settings and affect the consistency of treatment results.

Risk of complications: Although RFA is less invasive than surgery, complications can still occur, such as temporary voice changes or nodule rupture, underscoring the importance of experienced and careful procedural execution.

## 5. Safety and complications of RFA

RFA for BTNs is broadly recognized as a relatively safe treatment modality. However, identifying potential complications and implementing effective prevention and management strategies are essential to improve patient safety and clinical outcomes.

### 5.1. Complication rates and comparison with thyroidectomy

Although RFA is generally regarded as a safe and minimally invasive procedure, it still carries potential risks. The incidence of RLN injury during RFA ranges from 0.2% to 1.02%, with most cases involving transient symptoms that resolve spontaneously.^[26]^ Other complications, including hematoma formation and nodule rupture, are even less common, with reported rates between 0.1% and 0.5%.^[26]^

In contrast, traditional thyroidectomy is associated with a higher incidence of complications. Permanent RLN injury occurs in 2% to 5% of thyroidectomy cases, and postoperative hemorrhage is observed in 0.5% to 2% of cases.^[2]^ Furthermore, thyroidectomy frequently leads to permanent hypothyroidism, requiring lifelong thyroid hormone replacement due to the removal of functional thyroid tissue.^[2]^ These comparisons indicate that RFA has a notably lower complication rate than thyroidectomy, particularly regarding RLN injury and postoperative recovery. Nevertheless, achieving favorable outcomes with minimal risks requires careful patient selection and sufficient operator experience. RFA should be conducted by experienced clinicians with detailed knowledge of thyroid anatomy to minimize potential complications.

### 5.2. Possible complications and risk management strategies

Pain and swelling: Mild to moderate postprocedural pain and swelling are frequently observed but typically self-limiting. These symptoms can be effectively managed with cold compresses and non-prescription analgesics.^[23]^

RLN injury: Although rare, damage to the RLN may result in transient or permanent voice alterations. Preventive strategies include thorough anatomical evaluation before the procedure and the use of real-time ultrasound guidance to minimize the risk of injury to critical structures.^[24]^

Nodule rupture or bleeding: These rare complications are more likely to occur during ablation of large-volume nodules. Prevention includes accurate assessment of nodule size and location, along with meticulous control of energy output during the procedure.^[25]^

Endoscopic laryngeal evaluation in RFA procedures: A key safety measure in RFA for BTNs is the routine use of endoscopic laryngeal evaluation. Preoperative endoscopy is recommended for all patients, including euphonic individuals, to establish a baseline assessment of vocal cord and laryngeal anatomy.^[26]^ Postoperative endoscopy is essential in patients who develop vocal dysfunction following RFA. This approach enables early identification and intervention for RLN injury or other laryngeal complications, thereby reducing long-term sequelae and improving outcomes.

### 5.3. Limitation of RFA: absence of histopathological examination

A significant limitation of RFA is its inability to obtain histopathological samples, as the procedure ablates rather than excises the target tissue. Unlike surgical excision, which allows for comprehensive pathological analysis, RFA eliminates the possibility of post-procedural histological assessment. This limitation is particularly relevant when there is uncertainty regarding the potential malignancy of the nodule.^[17]^ In such cases, it is essential to inform patients of this diagnostic limitation and the potential need for surgery to obtain definitive histological confirmation.^[3]^ Clinicians should ensure that patients are fully aware of this limitation to support informed and shared decision-making. Although RFA is effective for benign nodules, it may not be appropriate for cases requiring definitive histopathological diagnosis.^[8]^

### 5.4. Reducing complication rates through technical improvements and procedure standardization

Recent advancements in RFA technology, such as finer electrodes and enhanced real-time imaging, have substantially improved procedural precision and safety. These improvements enable precise targeting and continuous monitoring, thereby reducing the risk of injury to adjacent tissues.^[19]^ The development and implementation of standardized operating protocols are essential for improving the safety of RFA. This includes comprehensive pretreatment evaluation, appropriate nodule selection, and adherence to technical guidelines. Furthermore, training and credentialing of operators are critical to minimizing complications and ensuring procedural success.^[17,24]^

A critical consideration is that RFA may complicate future thyroid surgery if surgical intervention becomes necessary. Studies indicate that surgery after RFA is often technically challenging due to fibrosis and scarring, which may increase surgical difficulty and the risk of complications, especially near critical structures such as the RLN.^[26]^ Therefore, patients should be counseled that although RFA is minimally invasive, it may pose challenges for future surgical procedures. Surgeons operating on patients with prior RFA should anticipate a more complex operative field and take extra precautions to reduce the risk of nerve injury and other complications.^[8]^

### 5.5. Enhancing complication management

Careful patient selection, considering nodule characteristics and overall health status, is essential for reducing the risk of complications. Thorough pre-procedural evaluations allow identification of high-risk individuals and the implementation of appropriate preventive strategies.^[18]^ A personalized follow-up plan is critical for the early detection and timely management of potential post-RFA complications. This should include regular imaging and functional assessments to monitor nodule regression and promptly address any emerging complications.^[21]^

## 6. Future prospects of RFA technology

The development of RFA technology is characterized by continual innovation and expanding clinical applications. Anticipated future advancements span several critical domains.

Enhanced precision and safety: Upcoming innovations will likely refine RFA devices and procedural techniques to enhance both precision and safety. These improvements include the incorporation of advanced imaging technologies to enhance visualization of target tissues and surrounding anatomical structures. Moreover, the development of intelligent, responsive probes or electrodes will support more accurate and controlled energy delivery, thereby reducing procedural risks and improving clinical outcomes.

Expanding indications: Ongoing research aims to broaden RFA’s clinical indications, including its use for diverse thyroid nodules and other benign or malignant conditions in different anatomical regions.^[26]^ This expansion seeks to optimize treatment protocols and extend RFA’s therapeutic potential across a wider range of conditions.

Combination therapies: Combining RFA with other therapeutic modalities, such as chemotherapy, immunotherapy, or targeted therapy, represents a promising strategy to improve overall treatment efficacy.^[3]^ These combined approaches may yield synergistic benefits, especially in treating larger or more aggressive tumors, thereby facilitating more comprehensive treatment strategies.

Minimizing complications: Technical advancements, improved patient selection, and standardized post-procedural care protocols are fundamental to reducing RFA-related complications.^[3]^ Ongoing improvements in these domains are essential for enhancing patient safety and ensuring procedural consistency.

Personalized medicine: Integrating molecular profiling and advanced imaging techniques will help tailor RFA interventions to individual patient characteristics. Such personalized strategies will incorporate genetic background, tumor biology, and prior treatment responses to develop tailored treatment plans that improve clinical efficacy and patient satisfaction.^[3,26]^

Integrated multidisciplinary care: RFA is anticipated to become an essential component of multidisciplinary care models, complementing surgery, radiotherapy, chemotherapy, and other interventions. This integration provides a minimally invasive option that enhances comprehensive treatment planning.^[26]^ The incorporation of RFA into multimodal regimens enables clinicians to address patient-specific needs more holistically. This collaborative approach may enhance tumor control, reduce treatment-related complications, and improve patients’ quality of life. Multidisciplinary collaboration enables the design of personalized treatment strategies, facilitating more effective disease control. Such strategies integrate tumor biology, patient preferences, and treatment goals to maximize therapeutic efficacy and improve long-term outcomes.

## 7. Summary

RFA has become a safe and effective minimally invasive option for treating BTNs, offering advantages such as faster recovery, fewer complications, and preservation of thyroid function compared to surgery and other thermal techniques. This study highlights key advances, including improved imaging and energy control for greater procedural accuracy, individualized treatment protocols based on nodule and patient characteristics, and the potential of combining RFA with other therapies to enhance efficacy. These innovations reflect a shift toward personalized, integrated care. Future research should aim to optimize RFA technologies, expand its clinical use, and embed it within multidisciplinary treatment frameworks. With ongoing refinement and the incorporation of personalized medicine, RFA is poised to play a central role in modern thyroid nodule management.

## Author contributions

**Conceptualization:** Zhuan-ning Han, Xu-ping Shen, Rong-rong Wang.

**Data curation:** Zhuan-ning Han, Rong-rong Wang.

**Investigation:** Rong-rong Wang.

**Methodology:** Zhuan-ning Han, Xu-ping Shen, Rong-rong Wang.

**Project administration:** Rong-rong Wang.

**Resources:** Zhuan-ning Han, Xu-ping Shen.

**Supervision:** Rong-rong Wang

**Validation:** Zhuan-ning Han, Xu-ping Shen, Rong-rong Wang.

**Visualization:** Zhuan-ning Han, Xu-ping Shen, Rong-rong Wang.

**Writing – original draft:** Zhuan-ning Han, Xu-ping Shen, Rong-rong Wang.

**Writing – review & editing:** Zhuan-ning Han, Xu-ping Shen, Rong-rong Wang.
